# Empowering Caregivers With Smart Care Artificial Intelligence

**DOI:** 10.1097/jnr.0000000000000727

**Published:** 2026-02-04

**Authors:** Shu-Ching Chen

**Affiliations:** School of Nursing, and Geriatric and Long-Term Care Research Center, College of Nursing, Chang Gung University of Science and Technology; School of Nursing, College of Medicine, Chang Gung University; Departments of Radiation Oncology and Proton Center, Chang Gung Memorial Hospital at LinKou, Taiwan, ROC

Caregivers play a vital role in continuum of care by providing essential physical, emotional, and social support to individuals with illness, disability, or ageing-related needs. The Organization for Economic Co-operation and Development (OECD) estimated the number of caregivers worldwide to exceed 350 million (OECD, 2023). The majority of care recipients are older adults living with chronic conditions, especially Alzheimer’s disease, cancer, stroke, cardiovascular disease, and diabetes (World Health Organization, 2023). The majority of caregivers are female (60–70%) and middle-aged (45–65 years old), with many needing to balance employment with family responsibilities (OECD, 2023). Burden of care associated with chronic conditions is associated with psychological distress, fatigue, and financial strain (Chan et al., 2023; Ping et al., 2025), and the convergence of ageing populations and rising chronic-disease prevalence underscores the urgent need for policies and technologies, including artificial intelligence (AI)-enabled smart care systems, to sustain caregiver well-being and care quality (Milella et al., 2023; Zhou et al., 2024).

Smart butler refers to intelligent home-management systems that integrate Internet of Things technologies, AI, and big-data analytics to support automated monitoring, decision-making, and personalized assistance in household settings (Sepasgozar et al., 2020). These systems use AI-driven behavioral analysis and contextual data to provide proactive reminders, detect anomalies, and enhance home safety, supporting independent living for older adults and reducing caregiver workload (Sisubalan et al., 2025). Virtual assistants and in-home robotic companions support medication adherence and daily routines, helping caregivers shift from continuous supervision to more efficient oversight (Lee et al., 2025; Maleki et al., 2025). Also, socially assistive robots may be programmed to deliver cognitive-impairment screening and early-warning assessments, enabling timely intervention and reducing burden (Luperto et al., 2025). In addition, smart-home functions such as virtual geofencing, bed-exit alarms, and voice-activated interfaces facilitate continuous monitoring to prevent wandering and falls (Kokorelias et al., 2024). The World Health Organization (2022) has highlighted the need to improve digital systems and enhance the capacity of older adults to use technology effectively, ensuring these tools can fully support caregivers and care recipients.

This issue of *The Journal of Nursing Research* features ten studies conducted in Taiwan, China, Turkey, and Indonesia that employ diverse research designs to address a broad spectrum of contemporary nursing and health-related concerns. The included articles encompass predictive modeling for frailty among community-dwelling older adults, the psychometric evaluation of a scale measuring understanding of complementary therapy use in diabetes, and an investigation of associations between traditional Chinese medicine body constitution and vertigo in women. Also, several intervention studies evaluate the effects of yoga on stress and coping self-efficacy among people living with HIV, the postoperative psychological consequences of fear and anxiety following open-heart surgery, and the impact of an Objective Structured Teaching Examination on clinical teaching efficacy in nurse preceptors. Additional contributions include a dual-path analysis of the influence of difficult nurse–patient relationships on compassion fatigue and satisfaction, the development and validation of an Indonesian version of a dementia-related health information access tool, and two meta-analyses synthesizing evidence on, respectively, nurses’ experiences of fragmented care in ageing populations and the performance of malnutrition-screening tools in individuals with chronic diseases. Together, the studies included in this issue offer valuable insights into clinical care, professional development, and population health; reflect methodological rigor; and advance nursing scholarship across diverse cultural and clinical contexts.

This is an open access article distributed under the Creative Commons Attribution License 4.0 (CCBY), which permits unrestricted use, distribution, and reproduction in any medium, provided the original work is properly cited.

*Address correspondence to: Shu-Ching CHEN; School of Nursing, College of Nursing, Chang Gung University of Science and Technology; Departments of Radiation Oncology and Proton Center, Chang Gung Memorial Hospital at LinKou, Taiwan, ROC. E-mail: shuching@gw.cgust.edu.twThe author declare no conflicts of interest.
